# Systematic review and meta-analysis of Chinese coach leadership and athlete satisfaction and cohesion

**DOI:** 10.3389/fpsyg.2024.1385178

**Published:** 2024-06-25

**Authors:** Jian Zhu, Meng Wang, Angelita Bautista Cruz, Hyun-Duck Kim

**Affiliations:** ^1^College of Physical Education, Keimyung University, Daegu, Republic of Korea; ^2^Department of Physical Education, Zhongyuan University of Technology, Zhengzhou, China

**Keywords:** Chinese coaches, multidimensional model of leadership in sport, leadership scale for sports (LSS), athletic satisfaction, group cohesion, coaching behaviors, player sex differences, amateur and professional athletes

## Abstract

**Introduction:**

This meta-analysis investigates the relationship between coach leadership behaviors and athlete satisfaction and group cohesion within the realm of Chinese sports. The study also explores player sex and player classification as potential moderating variables. The primary focus is on evaluating the impact of coaching behaviors, as measured by the Leadership Scale for Sports, on athlete satisfaction and group cohesion.

**Methods:**

Standard literature searches from China National Knowledge Infrastructure and Wanfang academic databases produced 26 studies encompassing a total of 319 effect sizes and a participant pool of 7,121 athletes across various sports.

**Results:**

Using the Comprehensive Meta-Analysis (CMA) to examine relevant data, results reveal a moderate and positive association between coach leadership and athlete satisfaction (ES = 0.412). Specifically, training and instruction (ES = 0.531), positive feedback (ES = 0.526), social support, and democratic decision-making exhibit positive effects, while autocratic behavior demonstrates a marginal positive effect. Similarly, a moderate positive relationship is identified between coach leadership and overall group cohesion (ES = 0.275), with training and instruction (ES = 0.396), social support (ES = 0.356), positive feedback, and democratic behavior positively influencing cohesion. Conversely, autocratic behavior has a small negative impact on cohesion. Furthermore, female athletes (ES = 0.603) and professional players (ES = 0.544) display stronger positive associations between coach leadership and satisfaction.

**Conclusion:**

These findings highlight the significance of diverse coaching behaviors aligned with player characteristics for fostering positive athlete satisfaction and group cohesion within the Chinese sports context, offering valuable guidance to Chinese coaches aiming to enhance their coaching strategies.

## 1 Introduction

Over the past several decades, China has emerged as a sports superpower. During the 1988 Seoul Summer Olympic Games, China ranked 11th with 5 gold, 11 silver, and 12 bronze medals. Subsequently, the country ascended to 4th place at both the 1992 and 1996 Olympic Games in Barcelona and Atlanta, respectively. Since then, China has consistently ranked among the top 3 in the overall medal standings at every Summer Olympic Games (International Olympic Committee, 2023). China’s dominance in sports is particularly evident in disciplines such as badminton, gymnastics, and volleyball (Zheng and Chen, 2016). Athletes in these disciplines often prevail in international competitions and major sporting events, such as the Olympics and World Championships. Their success is attributed to a combination of sport-specific factors, including government financial and policy support, access to superior facilities, and the opportunity to receive high-quality coaching and instruction (i.e., technical, tactical, and cognitive knowledge) from expert coaches (Zheng and Chen, 2016; Zheng et al., 2018).

Coaches in China, particularly those in elite sports, typically maintain direct contact with athletes, overseeing their holistic development. This involves creating a daily practice schedule to promote learning and improvement of sport-related skills through effective instructional strategies. Chinese coaches often assume additional roles as teachers and parental figures for athletes, especially because most professional athletes in China leave their homes at a young age to train and study in state-sponsored sports schools. Thus, coaches are responsible for overseeing athletes’ daily routines outside training, such as sleeping, eating, resting, and studying (Liu et al., 2022). Given the extensive time Chinese athletes spend with their coaches, both within and outside the training and competition environment, coaches inevitably play a critical leadership role in various aspects of the athletes’ sports careers and personal development. How coaches demonstrate these leadership behaviors can significantly impact athletes’ sports participation and overall well-being.

The leadership of coaches has garnered substantial attention from scholars in the sport psychology domain because of its profound impact on athletes’ sport-related outcomes (Gilbert and Trudel, 2004; Vella et al., 2010; Kim and Cruz, 2016, 2022; Cruz and Kim, 2017). Chinese scholars have recently shown considerable interest in exploring coach leadership as a research theme to gain deeper insights into the interactive dynamics between coaches and athletes, as well as the role of the coach as an influential contributor within the landscape of Chinese sports. Studies examining the effects of Chinese coaches’ leadership styles and behaviors on athletes’ psychological outcomes have identified both positive and negative associations (Lan, 2010; Zhu et al., 2017; Wang, 2021; Hsu, 2022; Zhang, 2022). Positive associations were observed when coaches displayed democratic or autocratic leadership styles and supportive behaviors, resulting in enhanced athlete satisfaction and cohesion (Jiao and Chen, 2007; Lan, 2010; Zhu et al., 2017; Tian et al., 2019; Hsu, 2022). Conversely, a negative association was observed when coaches employed autocratic leadership, leading to athletes’ dissatisfaction and reduced perceived cohesion (Jiao and Chen, 2007; Lan, 2010; Tian et al., 2019). These findings underscore the pivotal role of coach leadership styles and behaviors in shaping the psychological states of athletes within the Chinese sports setting. Consequently, for Chinese coaches to be effective sports leaders, they must be mindful of the behaviors they exhibit toward their players. These behaviors should aim to foster positive athletic experiences, ultimately contributing to desirable personal and sport-specific outcomes.

When examining the impact of coaches’ leadership behaviors on athletes’ sport-related outcomes, researchers frequently employ the Multidimensional Model of Sports Leadership (MMSL) (Chelladurai, 2007). The MMSL posits that an athlete’s satisfaction and performance depend on the required, actual, and preferred leader behaviors, which, in turn, can be influenced by situational, leader, and member characteristics.

To assess leadership behaviors using this model, Chelladurai and Saleh (2007) developed the Leadership Scale for Sports (LSS), comprising five subscales representing different components of sports leadership behaviors. These include instructional behaviors (training and instruction), decision-making behaviors (democratic and autocratic), and motivational tendencies (social support and positive feedback). Numerous studies have used this measurement tool to demonstrate that various factors can affect leader behavior, such as athletes’ nationality (Weinberg and Gould, 2015), age (Martin et al., 1999; Weinberg and Gould, 2015), sex (Cruz and Kim, 2017; Yenen et al., 2022), and type of sport (Terry, 1984; Yenen et al., 2022). Additionally, other studies have revealed various consequences related to leader behavior, such as performance (Horn, 2008; Moen et al., 2014; Perera, 2019), burnout (Price and Weiss, 2000), anxiety (Price and Weiss, 2000), motivation (Price and Weiss, 2000; Jin et al., 2022), cohesion (Gardner et al., 1996; Ronayne, 2004), and satisfaction (Riemer and Chelladurai, 1998; Ntomali et al., 2017; Fouraki et al., 2020; Jin et al., 2022). Indeed, the results of these studies support the MMSL framework, indicating a dynamic interplay among antecedent conditions, types of leadership behaviors, and sports outcomes. Furthermore, the evidence underscores the importance of the LSS in identifying and evaluating coaches’ leadership behaviors and determining which of these behaviors significantly affect the psychological and athletic outcomes of players.

A recent meta-analysis focused on coach leadership, primarily examining the MMSL framework and employing the LSS to assess the combined effects of coach leadership behaviors on athlete satisfaction and cohesion (Kim and Cruz, 2016). Athlete satisfaction is characterized as a positive emotional state resulting from athletes’ evaluations of their athletic experiences, encompassing structures, processes, and outcomes (Chelladurai and Riemer, 1997). By contrast, sports cohesion refers to individuals’ perceptions of the extent to which team members are united in pursuing sports objectives and goals (Carron, 1982). Their findings indicate that coaches’ overall leadership behaviors were moderately and strongly related to cohesion and satisfaction, respectively. Notably, the training and instruction dimension of leadership behavior exhibited the most substantial impact on both psychological outcomes.

Building upon the insights from this existing meta-analytic study by Kim and Cruz (2016), a systematic review and analysis of the empirical studies available in Chinese literature on sports leadership would be valuable, enriching the body of knowledge in sports leadership literature. Moreover, given the distinctive context of Chinese sports—where athletes often spend almost their entire lives in sports schools, training and studying exclusively under the rigorous and detailed supervision of coaches—exposure to commanding and firm coaching behaviors is generally deemed acceptable. Coaches are generally viewed as second parents by players (Li et al., 2015; Babbitt, 2019) and as the primary managers of athletes’ sports careers. Furthermore, due to the sport culture of the country that places greater expectations on winning as a function of sport level, athletes tend to trust their coaches more and rely on their authority and independent judgments concerning their athletic career. Likewise, since sport achievements and success are more valued, coaches are predisposed to adjust their coaching behaviors resulting from these expectancies (Amorose and Horn, 2000). Consequently, an authoritative leadership style may yield positive outcomes when such behavior is perceived as appropriate within the given context (Chelladurai, 2007). However, this assertion warrants further investigation.

This systematic meta-analysis consolidated relevant quantitative studies on sports leadership, evaluating the impact of coach leadership on athlete satisfaction and cohesion within the context of Chinese sports. Furthermore, the study examined player sex and sport classification as potential moderating variables in the relationship between coach leadership and athlete outcomes. By understanding the overall and specific dynamics of coach-athlete relationships in China, this meta-analytic review offers valuable insights for enhancing the leadership practices of Chinese coaches, particularly those behaviors that foster positive experiences in athletes’ athletic participation.

This study addressed the following research questions: (1) What is the overall effect size (ES) for the relationship between coach leadership and satisfaction? (2) What is the overall ES for the relationship between coach leadership and cohesion? (3) What are the ESs for the relationships between each dimension of coach leadership and satisfaction and cohesion? (4) Does player sex moderate the relationship between coach leadership behavior and athletes’ satisfaction and cohesion? (5) Does player sport classification moderate the relationship between coach leadership behavior and athletes’ satisfaction and cohesion?

## 2 Method

### 2.1 Literature search

**Figure 1:**
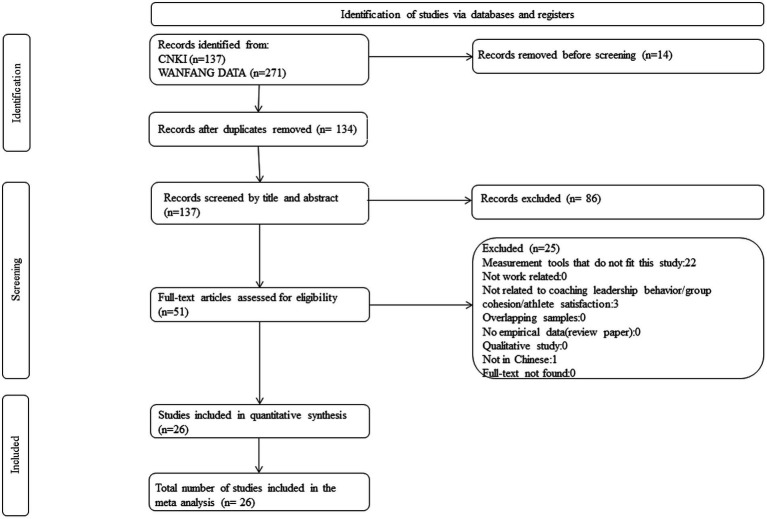
Flow diagram of study selection process following PRISMA procedure.

The authors conducted a systematic computer-based literature search and content analysis to collect relevant research studies addressing coach leadership behavior and target outcome variables following the PRISMA guidelines (Figure 1). The China National Knowledge Infrastructure and Wanfang academic databases were utilized to gather potential papers. The keywords used during the search process were leadership, coaching, coaches, leadership behavior, leadership sports scale, cohesion, (group) cohesion, and (athlete) satisfaction. Additionally, the authors manually searched for related articles in leading Chinese journals on sports psychology and sports management. The literature search covered the period from inception until April 2022. In this stage, a total of 408 articles were identified from the academic databases. Upon further screening and review articles, books, book chapters, conference proceedings and duplicates removed, 137 articles were considered qualified for further evaluation.

### 2.2 Inclusion criteria

To be included in the meta-analysis, studies should have (1) been published in academic journals; (2) employed a quantitative research design with Chinese sports coaches and athletes as participants; (3) evaluated coach leadership using the LSS; and (4) included relevant statistical variables such as correlation coefficient values and sample sizes to calculate the ES between coaching leadership behavior and satisfaction and/or cohesion. Studies that did not meet the inclusion criteria were excluded. Ultimately, 26 studies were accepted for inclusion in the meta-analysis.

### 2.3 Coding

Each study was reviewed and categorized based on its outcome variable(s). Two authors independently coded all relevant characteristics and statistical data from the studies. The third author reviewed the coding results and checked for discrepancies. The last author reassessed and rectified any observed coding discrepancies or errors. Finally, all authors reviewed the corrected coding list of studies for final confirmation.

A total of 319 ESs were coded from the 26 studies included in this meta-analysis. The sample comprised 7,121 participants, including both professional and amateur athletes from middle schools, high schools, colleges, and sports clubs. These participants were involved in a range of sports, including handball, basketball, soccer, volleyball, rowing, cheerleading, scuba diving, track and field, aerobics, and wrestling. Table 1 presents the characteristics of the included studies.

**Table 1:** Summary characteristics of Chinese coaching leadership studies using LSS.

#	Author and Year	Player sex	Player classification	Sample size	Type of sport	Dependent variable
1	Shi and Ji (2004)	Male	Amateur	165	Team	Satisfaction
2	Jiao and Chen (2007)	Female	Amateur	376	Team	Satisfaction
3	Zhang and Yin (2011)	Male	Amateur	207	Team	Satisfaction
4	Liao (2011)	Male/female	Amateur	130	No description	Satisfaction
5	Yan et al. (2012)	Male/female	Professional	184	Individual	Satisfaction
6	Cai and Wu (2014)	Male	Professional	339	Team	Satisfaction
7	Wang (2015)	Male	Amateur	136	Team	Satisfaction
8	Zhu et al. (2017)	Male/female	Professional	320	Team	Satisfaction
9	Wang (2018)	Female	Professional	196	Team	Satisfaction
10	Zhang (2019)	Male	Professional	274	Individual	Satisfaction
11	Liu (2021)	Female	Professional	283	Team	Satisfaction
12	Hsu (2022)	Male/female	Amateur	388	Team/ Individual	Satisfaction
13	Zhang (2022)	Female	Professional	438	Team	Satisfaction
14	Dai and Qiu (2022)	Male	Professional	304	Team	Satisfaction
15	Ma and Wang (2006)	Female	Professional	160	Team	Task and social cohesion
16	Zhang (2006)	Female	Amateur	581	Team/ Individual	Task and social cohesion
17	Zhu (2008)	Male	Professional	99	Team	Task and social cohesion/satisfaction
18	Zhang (2009)	Male/ female	Professional	343	No description	Task and social cohesion
19	Lan (2010)	Male	Amateur	465	Team	Task and social cohesion
20	Cui (2010)	No description	Professional	319	No description	Task and social cohesion
21	Li (2015)	Female	Amateur	218	Team	Task and social cohesion/satisfaction
22	Wu (2017)	Female	Amateur	297	Team	Task and social cohesion
23	Li (2017)	Female	Professional	167	Team	Task and social cohesion
24	Tian et al. (2019)	Male	Professional	144	Team	Task and social cohesion
25	Liu (2020)	Male	Professional	280	Team	Task and social cohesion
26	Wang (2021)	Female	Amateur	308	Team	Task and social cohesion

### 2.4 Meta-analytic tool

The Comprehensive Meta-Analysis (CMA) software (Borenstein et al., 2005) was utilized to calculate ESs from the selected studies. By converting the correlation coefficient and sample size from each study into Fisher’s *z* scale, the CMA software computed the summary effect. Additionally, the confidence interval (CI) results generated by the CMA indicated variability in the estimated mean correlation. Following the recommendation of Hunter and Schmidt (2004) when evaluating a relatively small number of studies, the random effects meta-analytic procedures were used. The resulting ESs were categorized as small (0.10), medium (0.30), and large (0.50) based on the benchmarks suggested by Cohen (1998).

## 3 Results

The potential for publication bias in studies concerning coach leadership, group cohesion, and athlete satisfaction was investigated (Supplementary Figure S1). The funnel plot for coach leadership and group cohesion studies displayed a symmetrical distribution of points on both sides, indicating no evidence of publication bias. Furthermore, Egger’s regression test yielded an intercept value of 0.445, with *p* > 0.05, corroborating the absence of publication bias.

The distribution of points in the funnel plot of studies on coach leadership and athlete satisfaction was somewhat asymmetric, suggesting possible publication bias. The Egger’s regression test intercept value was 0.448, *p* < 0.05, indicating potential bias. However, the Begg and Mazumdar rank correlation test yielded a value of 0.300, *p* > 0.05, suggesting no publication bias.

### 3.1 Relationship between coach leadership behavior and athlete satisfaction

The results showed a positive relationship between coach leadership and athlete satisfaction. The overall value of coach leadership concerning its effect on athlete satisfaction was computed as 0.412, suggesting that coach leadership has a moderate effect on athlete satisfaction (Table 2).

**Table 2:** Overall meta-analysis of coach leadership and athlete satisfaction.

*k*	*Q*	*p-value*	ES	−95% CI	+95% CI	I^2^	SE
16	1525.918	0.000	0.412	0.123	0.636	99.017	0.160

*k*, sample size; *Q*, heterogeneity test statistic; *p*, significance level of the heterogeneity test statistic; ES, weighted random effects size; CI, confidence interval; *I*^2^, ratio of the magnitude of total heterogeneity between studies to total variance; SE, standard error.

The effects of various leadership behaviors on athlete satisfaction were examined, with results indicating that training and instruction, as well as positive feedback, had large and positive impacts. By contrast, democratic behaviors and social support exhibited moderate and positive effects. Notably, although the mean ES of autocratic behavior was relatively small (0.074), its influence on athlete satisfaction was positive (see Table 3).

**Table 3:** Meta-analysis of the relationship between coach leadership dimensions and athlete satisfaction.

Leadership traits	*Q*	*p*	ES	−95% CI	+95% CI	*I*^2^	SE
Training and Instruction (*k* = 16)	1768.706	0.001	0.531	0.249	0.731	99.152	0.186
Democratic (*k* = 16)	1871.320	0.008	0.438	0.122	0.674	99.198	0.197
Autocratic (*k* = 16)	1740.644	0.667	0.074	−0.256	0.388	99.138	0.183
Social Support (*k* = 16)	1712.522	0.001	0.492	0.203	0.702	99.124	0.180
Positive Feedback (*k* = 15)	1159.965	0.000	0.526	0.288	0.703	98.793	0.130

*k*, sample size; *Q*, heterogeneity test statistic; *p*, significance level of the heterogeneity test statistic; ES, weighted random effects size; CI, confidence interval; *I*^2^, ratio of the magnitude of total heterogeneity between studies to total variance; SE, standard error.

### 3.2 Relationship between coach leadership behavior and group cohesion

The results illustrated a positive relationship between coach leadership and group cohesion. The overall value of coach leadership concerning its effect on group cohesion was computed as 0.275, suggesting that coach leadership has a relatively modest and positive effect on overall group cohesion (see Table 4).

**Table 4:** Overall meta-analysis of coach leadership and group cohesion.

Factor	*Q*	*p*	ES	−95% CI	+95% CI	*I*^2^	SE
Overall (*k* = 12)	84.496	0.000	0.275	0.184	0.362	86.982	0.013
Task (*k* = 12)	80.273	0.000	0.286	0.198	0.370	86.297	0.012
Social (*k* = 12)	100.328	0.000	0.264	0.164	0.358	89.036	0.015

*k*, sample size; *Q*, heterogeneity test statistic; *p*, significance level of the heterogeneity test statistic; ES, weighted random effects size; CI, confidence interval; *I*^2^, ratio of the magnitude of total heterogeneity between studies to total variance; SE, standard error.

In the subcategory of group cohesion, both task and social cohesion exhibited positive relationships with coach leadership, with values of 0.286 and 0.264, respectively. These findings suggest a positive and moderately significant impact of coach leadership on the task and social cohesion of athletes (see Table 4).

The relationship between each leadership behavior and overall cohesion revealed that, except for autocratic leadership behavior, all other leadership behaviors exhibited positive and moderate associations with group cohesion. Specifically, training and instruction demonstrated the most pronounced positive and moderate impact on group cohesion, followed by social support, democratic behavior, and positive feedback. Conversely, autocratic leadership had a negative effect on group cohesion, although the magnitude of this effect was minimal (see Table 5).

**Table 5:** Meta-analysis of the relationship between coach leadership dimensions and group cohesion.

Leadership dimension	Factor	Q	*p*	ES	−95% CI	+95% CI	*I*^2^	SE
Training	Overall	183.454	< 0.01	0.396	0.271	0.508	94.004	0.028
And Instruction	TC	191.376	< 0.01	0.423	0.298	0.533	94.252	0.03
(*k* = 12)	SC	212.936	< 0.01	0.368	0.231	0.491	94.834	0.033
Democratic	Overall	122.016	< 0.01	0.346	0.241	0.443	90.985	0.019
(*k* = 12)	TC	171.657	< 0.01	0.369	0.246	0.48	91.657	0.026
	SC	118.908	< 0.01	0.321	0.216	0.419	90.749	0.018
Autocratic	Overall	255.397	< 0.01	−0.051	−0.214	0.114	95.693	0.04
(*k* = 12)	TC	229.726	< 0.01	−0.051	−0.205	0.106	95.212	0.036
	SC	314.003	< 0.01	−0.052	−0.232	0.131	96.497	0.049
Social Support	Overall	125.008	< 0.01	0.356	0.25	0.453	91.201	0.019
(*k* = 12)	TC	169.939	< 0.01	0.308	0.181	0.425	93.527	0.026
	SC	270.435	< 0.01	0.398	0.246	0.532	95.932	0.042
Positive Feedback	Overall	161.785	< 0.01	0.298	0.173	0.413	93.201	0.025
(*k* = 12)	TC	206.894	< 0.01	0.343	0.205	0.468	94.683	0.032
	SC	138.105	< 0.01	0.251	0.133	0.362	92.035	0.021

*k*, sample size; *Q*, heterogeneity test statistic; *p*, significance level of the heterogeneity test statistic; ES, weighted random effect size; CI, confidence interval; *I*^2^, ratio of the magnitude of total heterogeneity between studies to total variance; SE, standard error; TC, task cohesion; SC, social cohesion.

The relationships of each leadership dimension with task cohesion and social cohesion revealed positive and moderate associations of training and instruction, democratic, social support, and positive feedback leadership behaviors with both task and social cohesion. Conversely, autocratic behavior displayed a negative, albeit very weak, association with both task and social cohesion (see Table 5).

### 3.3 Relationship between coach leadership, cohesion, and satisfaction based on player sex

The mean ES for female athletes was 0.603 while that for male athletes was 0.439. This suggests that both male and female athletes demonstrated positive relationships between coach leadership and satisfaction. However, the mean ES was higher in female athletes compared to their male counterparts (see Table 6).

**Table 6:** Coach leadership and athlete satisfaction relationship based on player sex.

Player sex	*Q*	*p*	ES	−95% CI	+95% CI	*I*^2^	SE
Female (*k* = 5)	1012.944	0.094	0.603	−0.118	0.908	99.605	0.635
Male (*k* = 7)	132.064	0.000	0.439	0.226	0.612	95.457	0.064

*k*, sample size; *Q*, heterogeneity test statistic; *p*, significance level of the heterogeneity test statistic; ES, weighted random effects size; CI, confidence interval; *I*^2^, ratio of the magnitude of total heterogeneity between studies to total variance; SE, standard error.

For the leadership-cohesion relationship, the mean ES for female athletes’ group cohesion, task cohesion, and social cohesion was 0.232, 0.239, and 0.225, respectively. These values indicate that the relationships between coach leadership and group, task, and social cohesion were positive with moderate effects. Similarly, the mean ES for male athletes’ group cohesion, task cohesion, and social cohesion was 0.383, 0.384, and 0.379, respectively, suggesting positive relationships between coach leadership and group, task, and social cohesion with moderate effects (see Table 7).

**Table 7:** Coach leadership and group cohesion relationship based on player sex.

Player sex	*Q*	*p*	ES	−95% CI	+95% CI	*I*^2^	SE
Female (*k* = 6)							
Overall	49.919	0.003	0.232	0.082	0.372	89.984	0.025
Task	49.896	0.002	0.239	0.089	0.379	89.979	0.025
Social	57.282	0.007	0.225	0.063	0.375	91.271	0.029
Male (*k* = 4)							
Overall	10.215	0.000	0.383	0.271	0.484	70.630	0.011
Task	13.284	0.000	0.384	0.256	0.498	77.416	0.018
Social	10.095	0.000	0.379	0.268	0.481	70.282	0.013

*k*, sample size; *Q,* heterogeneity test statistic; *p,* significance level of the heterogeneity test statistic; ES, weighted random effects size; CI, confidence interval; *I*^2^, ratio of the magnitude of total heterogeneity between studies to total variance; SE, standard error; TC, task cohesion; SC, social cohesion.

### 3.4 Relationship between coach leadership, cohesion, and satisfaction based on player classification

The relationship between coach leadership and athlete satisfaction was positive with moderate effects among amateur players (ES = 0.215). By contrast, coach leadership had a large and positive effect on athlete satisfaction among professional players (ES = 0.544) (see Table 8).

**Table 8:** Coach leadership and athlete satisfaction relationship based on player classification.

Player classification	*Q*	*p*	ES	−95% CI	+95% CI	*I*^2^	SE
Amateur (*k* = 7)	146.392	0.083	0.215	−0.028	0.434	95.901	0.068
Professional (*k* = 9)	1152.362	0.013	0.544	0.126	0.798	99.306	0.288

*k*, sample size; *Q*, heterogeneity test statistic; *p,* significance level value of the heterogeneity test statistic; ES, weighted random effect size; CI, confidence interval; *I*^2^, ratio of the magnitude of total heterogeneity between studies to total variance; SE, standard error.

The relationship between leadership and cohesion revealed positive and moderate associations between coach leadership and group cohesion (ES = 0.283), task cohesion (ES = 0.283), and social cohesion (ES = 0.283) among amateur athletes. Similarly, the relationships between coach leadership and group cohesion (ES = 0.269), task cohesion (ES = 0.288), and social cohesion (ES = 0.249) were positive with moderate effects among professional athletes (see Table 9).

**Table 9:** Coach leadership and group cohesion relationship based on player classification.

Player classification	*Q*	*p*	ES	−95% CI	+95% CI	*I*^2^	SE
Amateur (*k* = 5)							
Overall	54.145	0.001	0.283	0.120	0.431	92.612	0.028
Task	48.689	0.000	0.283	0.128	0.424	91.785	0.025
Social	68.920	0.003	0.283	0.098	0.449	94.196	0.035
Professional (*k* = 7)							
Overall	29.549	0.000	0.269	0.159	0.373	79.695	0.014
Task	31.414	0.000	0.288	0.176	0.394	80.901	0.015
Social	29.505	0.000	0.249	0.138	0.354	79.664	0.014

*k,* sample size; *Q*, heterogeneity test statistic; *p*, significance level of the heterogeneity test statistic; ES, weighted random effects size; CI, confidence interval; *I*^2^, ratio of the magnitude of total heterogeneity between studies to total variance; SE, standard error; TC, task cohesion; SC, social cohesion.

## 4 Discussion

This study aimed to comprehensively examine the body of research focused on the impact of coach leadership, as measured by LSS on athlete satisfaction and group cohesion within the context of Chinese sport. Additionally, the study investigated players’ sex and sports classification as moderating variables for the associations between the predictor and outcome variables. Overall, results revealed that the magnitude and direction of the relationship between the predictor and outcome variables are moderate and positive. Furthermore, player characteristics such as sex and sport classification were found to moderate the coach leadership-satisfaction/cohesion relationships.

### 4.1 Relationship between coach leadership and satisfaction

Overall, coach leadership was moderately and positively associated with athlete satisfaction. This result is relatively similar to that of a previous meta-analysis (Kim and Cruz, 2016), which also revealed a moderate association between coach leadership and satisfaction. However, the mean ES of the present study is slightly higher than that of the previous one (ES = 0.412 vs. ES = 0.357).

In terms of various dimensions of leadership behavior, the results indicated that all five dimensions are positively associated with athlete satisfaction, corroborating the findings of a previous study (Kim and Cruz, 2016). Specifically, training and instruction (ES = 0.531) and positive feedback (ES = 0.526) behaviors were found to exert large effects on athlete satisfaction. Social support and democratic behaviors demonstrated moderate effects, while autocratic behavior had a negligible effect. Although these results partially confirm the findings of a previous meta-analysis (Kim and Cruz, 2016), the current study revealed larger effects for training and instruction and positive feedback behaviors compared to the results of the earlier study (Kim and Cruz, 2016), which indicated only moderate effects for both behaviors (ES = 0.432 and 0.398, respectively). The study’s inclusion criteria, particularly the exclusive sample population, could account for the discrepancy in results.

The results highlight that various training, decision-making, and motivational-related behaviors of coaches can affect the satisfaction levels of athletes, with training and instruction and positive feedback being the largest contributors to the leadership-satisfaction relationship. Therefore, the findings imply that Chinese coaches should extensively focus on delivering high-quality training and instruction to athletes and offer frequent positive feedback on their performance to enhance athletes’ overall satisfaction with their athletic experience. Furthermore, Chinese coaches should encourage players to make decisions independently, provide relational support, and exercise control to instill discipline and foster long-term commitment to sports among athletes.

### 4.2 Relationship between coach leadership and cohesion

The results indicate that the overall magnitude of the relationship between coach leadership and group cohesion is moderate. Similarly, both types of group cohesion—task cohesion (ES = 0.286) and social cohesion (ES = 0.264)—demonstrated positive and moderate associations with coach leadership. These findings corroborate those of a similar meta-analytic study, which reported moderate ES values for the relationships between leadership and group cohesion (ES = 0.211), task cohesion (ES = 0.221), and social cohesion (ES = 0.201) (Kim and Cruz, 2016).

The association between each leadership dimension and group cohesion, including its subtypes, revealed that all behaviors—except for autocratic behavior—such as training and instruction, democratic, social support, and positive feedback, have a positive relationship with a moderate effect on the group, task, and social cohesion. These findings align with a previous meta-analysis that examined leadership dimensions of coaches using the LSS (Kim and Cruz, 2016). Notably, ESs in the current study are slightly higher than those reported in the earlier meta-analytic review. This suggests that when Chinese coaches frequently exhibit these leadership behaviors, players tend to feel more cohesive with their teammates and are more committed to achieving team goals.

By contrast, a negative correlation with a negligible ES is observed between autocratic behavior and group, task cohesion, and social cohesion. This suggests that a player’s commitment to achieving the team’s goal and interpersonal attraction with teammates tend to decrease when coaches are perceived as overly controlling, intimidating, and unsympathetic. However, crucial to note is not dismissing the possibility that autocratic behavior has any (negative) relationship with cohesion. This caution stems from the CI, encompassing the 0% null difference (Greenland et al., 2016), as observed in an earlier study (Kim and Cruz, 2016). Therefore, interpreting this result requires caution, and additional empirical studies are warranted to further explore the relationship between autocratic behavior and group cohesion, including its subtypes.

### 4.3 Relationship between coach leadership and athlete satisfaction and cohesion based on player sex

Previous studies have demonstrated that member characteristics can moderate the relationship between coach leadership and satisfaction (Kim and Cruz, 2016, 2022). Regarding player sex, coach leadership was positively associated with satisfaction in both males (ES = 0.439) and females (ES = 0.603); however, the relationship was stronger in females. This indicates that female athletes may experience greater satisfaction from positive coaching behaviors than male athletes.

Athletes’ overall cohesion and cohesion subgroups demonstrated a positive association with coach leadership for both male and female players. Furthermore, although the magnitudes of the associations were moderate, the mean ES value was slightly higher in male than in female athletes. This suggests that the perception of unity to accomplish tasks and interpersonal attraction might be slightly better in male athletes than in female athletes when coaches exhibit positive leadership behaviors.

The findings corroborate previous research outcomes demonstrating that the positive relationship between coach leadership and both satisfaction and cohesion varies according to player sex (Kim and Cruz, 2016, 2022). Notably, in the current study, the influence of coach leadership on satisfaction and cohesion for both male and female athletes appears more pronounced than in the earlier systematic review. In the previous meta-analysis study, Kim and Cruz (2016) observed ESs for the leadership-satisfaction in male and female athletes were 0.424 and 0.174, respectively. Whereas the ES for the leadership-cohesion relationship was 0.193 for male players and 0.174 for female players. Variations in study samples may have contributed to the disparities in the results between these studies, with the previous study primarily composed of studies conducted in western countries. This finding implies that there are notable sociocultural variations in how athletes perceive the behaviors exhibited by their coaches and how these behaviors impact athletes’ sport-related outcomes, as well as provides additional support that athletes’ culture or nationality as an important antecedent of leadership.

### 4.4 Relationship between coach leadership and athlete satisfaction and cohesion based on player classification

Results revealed that the positive association between coach leadership and satisfaction levels is stronger among professional athletes than among their amateur counterparts (ES = 0.544 vs. ES = 0.215). A previous study indicated that elite athletes prefer more democratic and social support behaviors than club athletes. Conversely, club athletes favored coaches who often display rewarding behavior, a pattern observed less frequently among elite players (Terry, 1984). Another study found that athlete satisfaction varied between senior and junior levels, with senior athletes reporting greater satisfaction when perceiving coaches as frequently providing training and instruction, as well as social support behaviors (Ntomali et al., 2017). In this context, when Chinese coaches adapt their behavior appropriately, aligning with the preferences of the athletes, satisfaction is more likely to develop. This alignment supports the notion that athlete satisfaction is linked to congruence between leader behaviors and athlete preferences (Riemer and Toon, 2001; Kao et al., 2015). It further supports the MMSL framework, which posits that athlete sport-related outcomes (i.e., satisfaction) depend on the dynamics between leader behavior and antecedent conditions (Chelladurai, 2007).

The relationships between coach leadership and group cohesion, as well as its subgroups, demonstrated positive correlations, yielding similar moderate ESs for both amateur and professional players. These results suggest that coaching behaviors, as defined by the LSS scale, can moderately influence athletes’ perceptions of cohesion, regardless of whether the players participate at the amateur or professional level. The similar effect sizes for the leadership-cohesion relationships based on sport classification may have resulted from the sample participants who are mostly team sport players representing particular geographical areas or state-sponsored sport schools. As previously mentioned, Chinese athletes generally enter sports schools at a very young age and spend their lives mastering sport-related knowledge and skills with the goal to participate in sport competitions and achieve sporting success. For example, professional athletes are those who are selected and trained by local teams with a primary goal to participate in city games, provincial games, national games, and other institutional competitions. They are fully supported by the state and relatively stable in terms of membership. Professional athletes also receive wages while their competition and training expenses are backed by the government. In this case, it is possible that Chinese athletes, regardless of sport classification, are likely to perceive higher level of cohesion due to similarities in competitive environment, group norms, group tasks, attitudes, cognition, and motives with regard to their athletic participation. This notion supports not only the MMSL model (Chelladurai, 2007) but also Carron (1982) which suggests that aside from leadership factors (i.e., leadership behaviors), task and social cohesion can result from environmental, personal, and team factors. Hence, the present findings highlight that better coach leadership is related to better cohesion in both amateur and professional athletes. Therefore, coaches should not only focus on leadership strategies that would enhance task cohesion among athletes, but also introduce interventions that promote compatibility and interpersonal closeness between leader and members.

Generally, the findings underscore the significant roles of player sex and classification in elucidating the relationship between coach leadership and athlete satisfaction and group cohesion. Furthermore, the current results enrich the sports leadership literature by synthesizing pertinent studies and performing a meta-analysis on the impact of coaches’ leadership behaviors on athletes’ sport-related outcomes within the Chinese context.

### 4.5 Limitations and future directions

This meta-analysis investigated only coaching behaviors and their effects on athlete satisfaction and group cohesion within the Chinese context. Consequently, the findings are generalizable solely to this population. Therefore, further research must be conducted on coach leadership and sport-related outcomes among athletes from diverse countries. Should sufficient studies become available, aggregating and categorizing the data by country could yield a more accurate understanding of whether the dynamics of coach leadership and sport-related outcomes are consistent across various national or cultural contexts, or if unique factors may influence the generalizability of the results.

Another limitation is the restriction of the measurement tool to the five dimensions defined in the LSS scale. Given that the LSS scale is the widely used tool in evaluating leadership of sport coaches based on the MMSL model, it is just appropriate to use this leadership measurement scale. Moreover, researchers examining coaches’ leadership in the context of Chinese sports generally employed this measurement tool in their studies, and in turn, produced adequate number of articles to conduct meta-analysis Consequently, inferences about the impact of coaching behaviors on athlete satisfaction and cohesion might differ if alternative tools were used to evaluate coaches’ leadership. Therefore, consolidating sports leadership studies that have utilized other prominent scales, such as the Coaching Behavior Scale for Sport (Côté et al., 1999), Multifactor Leadership Questionnaire (Bass and Avolio, 1997), and Differentiated Transformational Leadership Inventory (Callow et al., 2009) would be beneficial. This would provide better insights into which leadership styles and behaviors are more effective in fostering positive outcomes among athletes, beyond those assessed by the LSS scale or if the type of measurement tool acts as a moderator between the leadership-athlete satisfaction and team cohesion relationships.

### 4.6 Practical implications

Based on the findings of the study, for Chinese athletes to feel more satisfied with their athletic experiences as well as to perceive that their team are united in achieving their sport goals and objectives, and consequently lead to successful sport performance, Chinese coaches should display positive leadership behaviors that are also congruent to the personal characteristics of the players. For example, coaches of team sports in China dealing with female as well as elite (professional) athletes should frequently provide detailed and high-quality instructions focused on physical and mental development based on athletes’ specific positions within the team. Game strategies should also be properly explained to the team and make sure that these strategies are extensively practiced to attain a high level of mastery. In this way, team players would be able to execute the appropriate movement patterns quickly and accurately against their opponents in sport competitions.

Offering feedback that is both motivational and instructional is another leadership behavior that coaches should consider implementing when interacting with athletes. This feedback should focus on providing quality information, such as describing performances that meet set standards successfully or identifying movement skills or behaviors that athletes themselves also acknowledge as needing modification for their athletic development. This leadership approach when properly provided to athletes during training and competitive situations, and at the same time recognized by athletes to be relevant and helpful for their athletic improvement is likely to increase athletes’ positive emotions, leading to positive consequences such as enhanced motivation, competence, satisfaction and performance (Allen and Howe, 1998; Carpentier and Mageau, 2016; García-Herrero et al., 2022).

Meanwhile, as the present findings showed autocratic behavior to negatively impact both task and social cohesion, Chinese coaches should avoid controlling behaviors or create a training environment that is too rigid and structured. For example, despite the highly competitive nature of Chinese sports and the expectation for athletes to achieve performance success, coaches need to demonstrate flexibility in exercising their authority based on the competitive season or situation. For instance, Driscoll (2000) reported that successful team athletes expected their coach to be authoritative to maintain their focus, direction, and intensity. It was also observed that athletes acknowledged that coach yelling was acceptable during practice, especially when team members’ efforts were below par. In contrast, constant yelling and screaming by coaches at athletes during game situations were perceived as undesirable and ineffective coaching behavior. Hence, coaches should be mindful of the training and competitive situations when displaying autocratic behaviors toward athletes because these may facilitate or undermine levels of team cohesion.

## 5 Conclusion

This meta-analysis revealed that athletes’ sport-related outcomes, particularly athlete satisfaction and group cohesion, can be shaped by the leadership behaviors of their Chinese coaches. Specifically, leadership behaviors that emphasize training and instruction, positive feedback, social support, and democratic decision-making are associated with moderate to high levels of satisfaction and group cohesion among Chinese athletes. Conversely, autocratic behaviors displayed by Chinese coaches may slightly increase athlete satisfaction but negatively impact group cohesion. Additionally, leadership behaviors of Chinese coaches are linked to higher satisfaction levels among female and amateur athletes compared to male and professional athletes. Moreover, these leadership behaviors positively and moderately influence group cohesion for both male and female players, as well as amateur and professional athletes. Consequently, for Chinese coaches to be effective sports leaders, they should exhibit diverse coaching behaviors that align with the players’ characteristics and the sport’s sociocultural environment.

## Data availability statement

The raw data supporting the conclusions of this article will be made available by the authors, without undue reservation. Requests to access these datasets should be directed to JZ, 5418@zut.edu.cn.

## Author contributions

JZ: Software, Resources, Formal analysis, Writing – review & editing, Writing – original draft, Conceptualization. MW: Software, Resources, Writing – review & editing, Formal analysis. AC: Writing – original draft, Conceptualization, Writing – review & editing. H-DK: Supervision, Writing – review & editing, Writing – original draft, Software, Methodology, Formal analysis, Conceptualization.
