# Supplementary figures and images for: Systematic review and meta-analysis of Chinese coach leadership and athlete satisfaction and cohesion
Source: Front Psychol. 2024 Jun 25;15:1385178. doi: 10.3389/fpsyg.2024.1385178 (PMC11232180; doi:10.3389/fpsyg.2024.1385178)

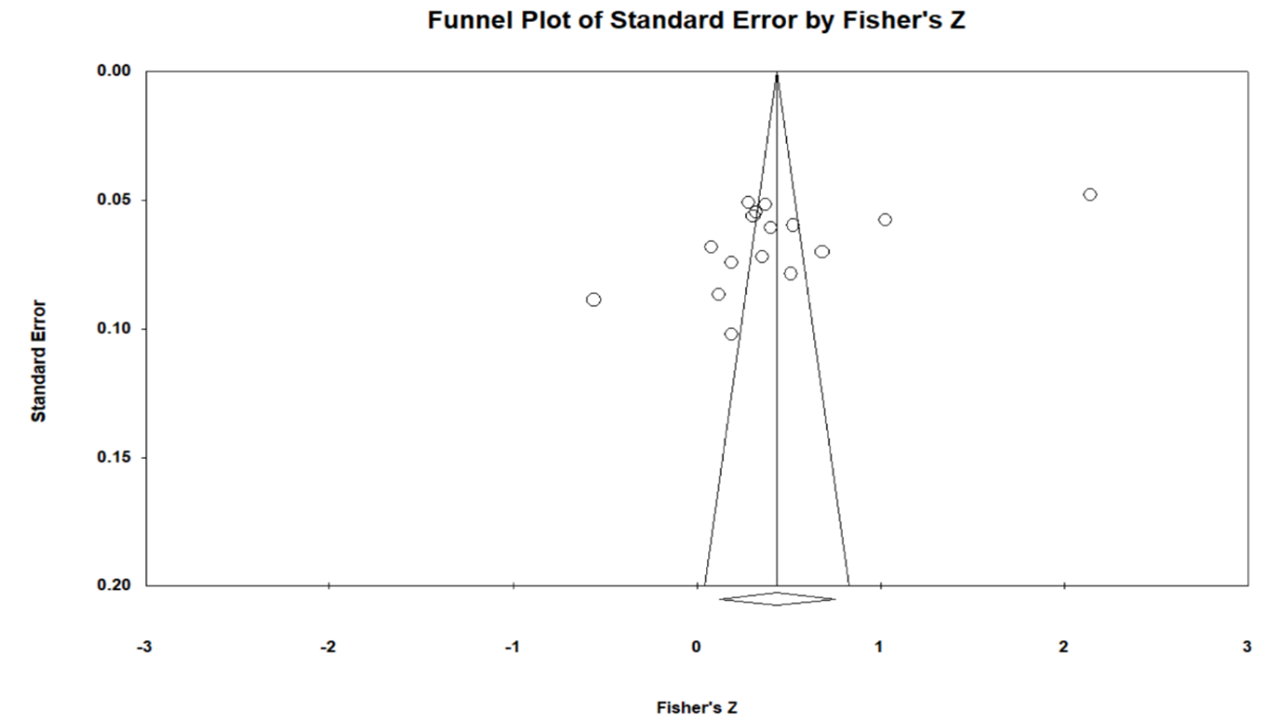

Supplement: Supplementary file 1 [file Image_1.TIF]
